# Multimodal Chemosensory Integration through the Maxillary Palp in *Drosophila*

**DOI:** 10.1371/journal.pone.0002191

**Published:** 2008-05-14

**Authors:** Takashi Shiraiwa

**Affiliations:** Department of Molecular, Cellular and Developmental Biology, Yale University, New Haven, Connecticut, United States of America; Freie Universitaet Berlin, Germany

## Abstract

Drosophila melanogaster has an olfactory organ called the maxillary palp. It is smaller and numerically simpler than the antenna, and its specific role in behavior has long been unclear. Because of its proximity to the mouthparts, I explored the possibility of a role in taste behavior. Maxillary palp was tuned to mediate odor-induced taste enhancement: a sucrose solution was more appealing when simultaneously presented with the odorant 4-methylphenol. The same result was observed with other odors that stimulate other types of olfactory receptor neuron in the maxillary palp. When an antennal olfactory receptor was genetically introduced in the maxillary palp, the fly interpreted a new odor as a sweet-enhancing smell. These results all point to taste enhancement as a function of the maxillary palp. It also opens the door for studying integration of multiple senses in a model organism.

## Introduction

When animals evaluate food, potential danger or other environmental cues, they utilize information which comes in many forms; smell, taste, sound, touch and vision. All these sensory inputs, including those stored as memory, are combined and lead to the appropriate behavioral response. The processing of multisensory information has attracted much attention. Successful approaches to this problem have been through observation of animal behavior, electrophysiology on cats, monkeys and mice, and human psychology [1], [2]. Of the possible sensory pairs for cross-modal integration, olfaction and taste have been particularly well described in human psychophysical studies. Sweet, bitter, sour, salty and umami are the only human taste modalities; others are olfactory but confused as taste [3]. Taste enhancement through olfaction is another example of the integration of these senses. Sweetness is enhanced by strawberry or lemon odor, but not by peanut butter or ham odor [4], [5]. The pairing of certain smells and tastes suggests the existence of a pre-defined neuronal network bringing the two senses together. It would be very advantageous to study sensory integration in a model organism amenable to genetic analysis.

*Drosophila melanogaster* has two olfactory organs, the third segment of the antenna and the maxillary palp [6]. They are both covered with hair-like structures called sensilla which harbor olfactory sensory neurons (OSNs). Olfactory sensilla are classified into three types by their morphology: basiconic, coeloconic and trichoid are found on the antenna while basiconic sensilla are the only olfactory sensilla on the maxillary palp. Compared to the maxillary palp, the antenna has 10 times more OSNs (1200 : 120) [7] and about 6 times more OSN types (38 : 6) [8], [9]. Some antennal OSNs have specialized functions such as pheromone reception in the trichoid sensilla or CO_2_ detection in the ab1C OSN which results in avoidance behavior [10]–[14]. Olfactory receptor genes are expressed in OSNs, which project to the glomeruli in the antennal lobe [15]–[18]. Projection neurons forward the information to the calyx of the mushroom body and the lateral horn [19]–[21]. This information relaying system is very similar in vertebrates where OSNs project to the glomeruli in the olfactory bulb and synapse with the mitral/tufted cells which then convey the input to the olfactory cortex. The taste system in the fly is thought to have a similar relaying system. The gustatory receptor neurons from the labellum project to the subesophageal ganglion (SOG) before being sent to higher order structures of the brain [22]–[26].

In this study the effect of odor on taste behavior through the maxillary palp was examined. In the presence of odors, antenna-less flies responded to a lower concentration of sucrose. Stimulation of every type of OSN in the maxillary palp had an identical effect. Which odors enhance taste depends on which olfactory receptors are expressed in the maxillary palp. This was confirmed by ectopic expression of an olfactory receptor, which resulted in an altered taste behavior. Ablation of the mushroom body, a neural locus involved in olfactory memory [27]–[29], did not alter taste enhancement. Furthermore, newly eclosed flies that never tasted food as adults still displayed odor-induced taste enhancement. The taste enhancement function of the maxillary palp was conserved in *D. simulans, D. pseudoobscura*, and *Musca domestica* (the house fly).

## Results

### OSNs in the maxillary palp are involved in taste enhancement

**Figure 1:**
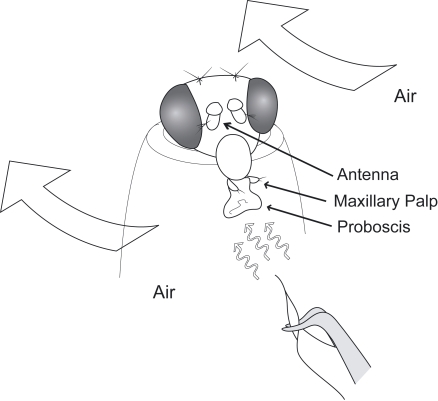
Fly immobilized for modified PER assay. The fly is placed into a truncated yellow tip. Tastant is presented to the fly with a piece of paper dipped in a sucrose solution.

In addition to the antenna, *Drosophila melanogaster* has an additional olfactory organ, the maxillary palp (Fig. 1). There has been little explanation for the existence of this secondary organ. It has been shown that if the antenna were removed, leaving the maxillary palp as the only olfactory organ, the avoidance behavior to high concentrations of chemicals is drastically reduced to 1/10–1/100 of that of an intact fly [30], [31]. It seems unlikely that a second olfactory organ exists just to provide such a small contribution to the animal's behavior. Because of its proximity to the proboscis and its neat packaging inside the head capsule when the proboscis is not extended, it seems likely that the maxillary palp is involved in feeding behavior. When the proboscis extends, the club-shaped maxillary palp is held at a 90-degree angle to the proboscis, as if it is designed for maximum exposure to any odor emanating from the food. In order to test this hypothesis, the feeding behavior with and without an additional odor was investigated. First, the proboscis extension response (PER) to various concentrations of sucrose was measured; the same was done in the presence of an odor, 4-methylphenol. PER indicates how appealing a taste (or taste in combination with an odor) is to the fly. 4-methylphenol was chosen because it elicits a robust electrophysiological response in the maxillary palp [32].

**Figure 2:**
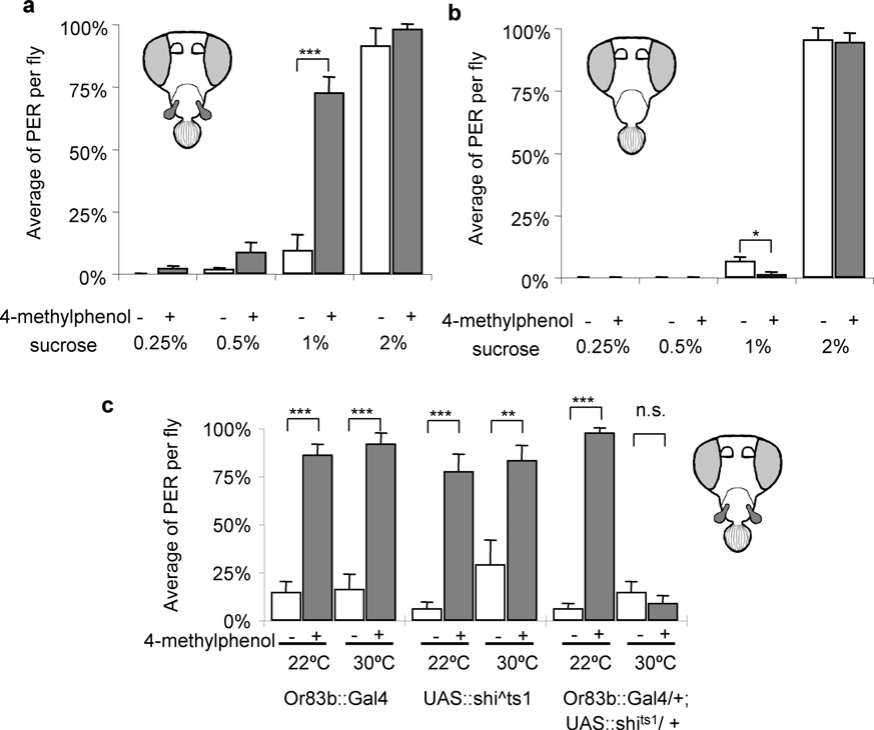
Effect of an odorant 4-methylphenol on PER. Wild type flies without a) antenna (Ant ^−^, MP+), b) no olfactory organs (Ant ^−^, MP ^−^) are tested. c) Antenna less flies (Ant ^−^, MP+) of Or83b::Gal4, UAS::shibire^ts1^ and the progeny from crossing these two lines (Or83b::Gal4×UAS::shibire^ts1^) were tested with 1% sucrose. +/− indicates the presence/absence of 4-methylphenol (0.2% v/v) in the sucrose solution. For all figures: Error bars are S.E.M. n = 7 to 14. Paired stutdent's *t-*test were done on arcsin converted values. * : p<.05, ** : p<.01, *** : p<.001.

Starved antenna-less flies displayed a large PER (91%) to 2% sucrose solution, but with 1% sucrose the responses were very small (3%) (Fig. 2a, Movie. S1). When 4-methylphenol was added to the solution, the PER to 1% sucrose was increased to 72%. The addition of 4-methylphenol did not have a large effect when the sucrose concentration was lower or higher than the detection threshold. This suggests that the information sent to the brain from the olfactory organ is supplementary to taste and that odor alone cannot evoke the proboscis extension.

To confirm that the effect of 4-methylphenol was mediated through olfaction and not taste, the olfactory input from the maxillary palp was removed by two methods; surgical removal of the maxillary palp and expression of *shibire^ts1^* in the maxillary palp. Flies without both olfactory organs (Ant ^−^, MP ^−^) did not display any increase in PER, though a slight reduction was observed, probably because of the unpleasant taste of 4-methylphenol mixed in the solution (Fig. 2b). *shibire^ts1^* is a semi-dominant temperature sensitive allele of dynamin, a protein which is crucial for synaptic vesicle recycling [33], [34]. Shifting the fly to a non-permissive temperature (>29°C) allows disruption to synaptic transmission in cells expressing this allele. The promoter region of *Or83b*, an olfactory receptor expressed in all OSNs, was used to drive expression of *shibire^ts1^* in the maxillary palp [17], [35]. Although this promoter drives expression in both olfactory organs, the effect on the maxillary palp can be measured by removing the antenna. The increase in PER with 4-methlphenol disappeared when temperature was shifted from 22°C to 30°C in flies that expressed *shibire^ts1^* in the maxillary palp (Fig. 2c). The same temperature shift did not have effect in the parental lines (*Or83b*::Gal4 and UAS:: *shibire^ts1^* ).

**Figure 3:**
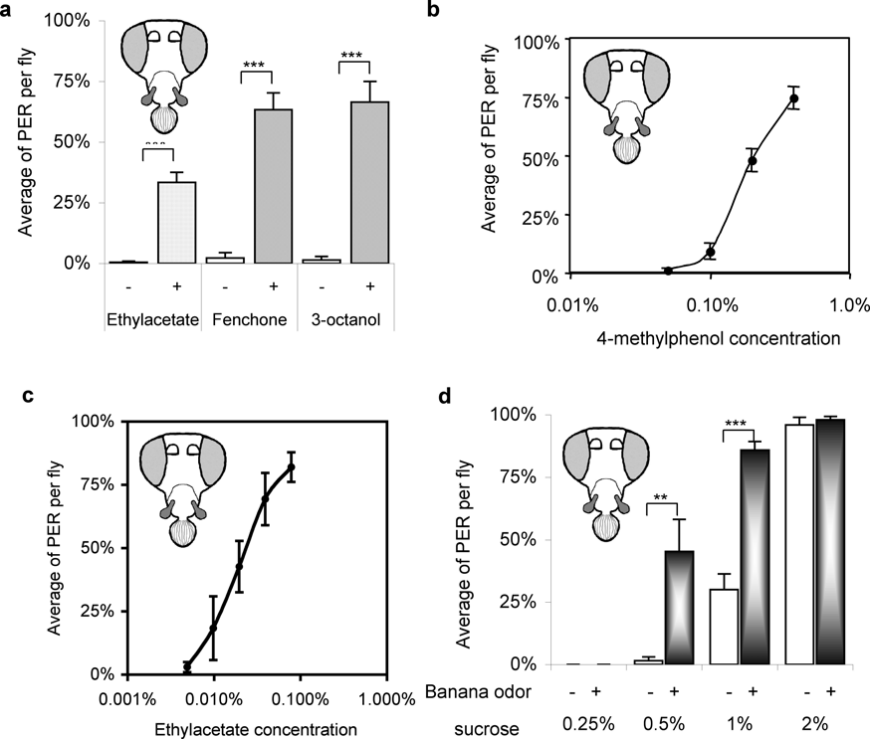
Taste enhancement through the maxillary palp by various odors. a) The effect of ethyl acetate (0.01%, mixed together in sucrose solution), Fenchone and 3-octanol (pure liquid deliver by a cotton swab) were tested. The sucrose concentration was 1% for all samples. The addition of these odors did not have a significant effect at other sucrose concentrations (data not shown). Ant ^−^, MP^+^flies were used. b) Dose response relationship of taste enhancement in the maxillary palp with 4-methyphenol and c) ethyl acetate. Average PER from Ant ^−^, MP^+^flies is plotted. The sucrose concentration was 1% for all samples. The X-axis is on a log scale. d) Effect of banana odor on taste. Overripe banana was used as odor source instead of pure chemicals. Ant ^−^, MP^+^flies were used. n = 7 to 14

There are six different types of OSNs in the maxillary palp, each with a different electrophysiological response profile to odors [32]. 4-methylphenol elicits a response only from 2 out of 6 OSNs, the pb1B and pb2B neurons. Is the odor-induced taste enhancement a general function of the maxillary palp or a phenomenon driven just by these two types of neurons? In order to answer this question I tested odors that stimulate the other four neuronal types. Ethyl acetate (0.01%), fenchone and 3-octanol were selected since these odors are the best stimuli for pb1A (ethyl acetate), pb2A (fenchone), pb3A (3-octanol) and pb3B (3-octanol) [32], [36]. Removing the antenna makes it possible to measure the effect of these odors on taste behavior through the intended neurons in the maxillary palp. All of these odors evoked a response similar to that observed with 4-methylphenol, and had a significant effect on the PER at a sub-threshold concentration of 1% sucrose (Fig. 3a). This shows that taste enhancement is a function of the maxillary palp and multiple types of neurons are involved in it.

The dose-response relationship of this maxillary palp-induced taste enhancement was measured (Fig. 3 b, c) for 4-methylphenol and ethyl acetate, which are odors that can be dissolved in the sucrose solution. The sigmoid dose response curve spans only around a ten-fold concentration range. This small concentration range should be very suitable for examining the odor of a food immediately in front of the fly and would not be affected by a faint scent emanating from a distance source. The enhancement of taste was observed only at relatively high concentrations of odors. Fenchone and 3-octanol are nearly insoluble in water, so pure liquid was presented to the fly with a cotton swab, and the highest concentration used for 4-methylphenol was close to the maximum concentration possible in water. Under these conditions the fly should experience an intense degree of smell, which yields enhancement, as opposed to inhibition of response. In behavioral assays, it has been shown that *Drosophila* avoids extremely high concentrations of odors that are attractive at low concentration [37]–[39]. This finding indicates that the maxillary palp does not convey negative information that prevents the fly from making contact with the food source.

The chemical concentration of odorants in nature is lower and the composition is more complex than those tested here. In order to assess the biological relevance of these findings, I asked whether a natural food source is capable of inducing taste enhancement through olfaction. The odor of an overripe banana was capable of enhancing taste to a level similar to the pure odorants used in this study (Fig. 3d). Ethyl acetate is one of the major volatile compounds in banana [40], and also an odor that enhances taste through olfaction in the maxillary palp (Fig. 3a). Other complex odor sources, such as beer, yeast paste, cheese and soy sauce were tested, but those did not display enhancement of taste (data not shown).

### Taste-smell association: learned or hard-wired?

In human psychological studies, it has been shown that perceptually similar smells and tastes, such as “sweet smell”, will enhance each other when presented together. The sweetness of sucrose is enhanced by strawberry odor and the detection threshold of benzaldehyde (a cherry/almond odor) is lower when saccharin stimuli was applied [4], [41]. It is unclear how certain smells and tastes are associated with each other. It would be very interesting to know if the association is formed through experience or is genetically specified. How did 4-methylphenol become associated with sweetness?

**Figure 4:**
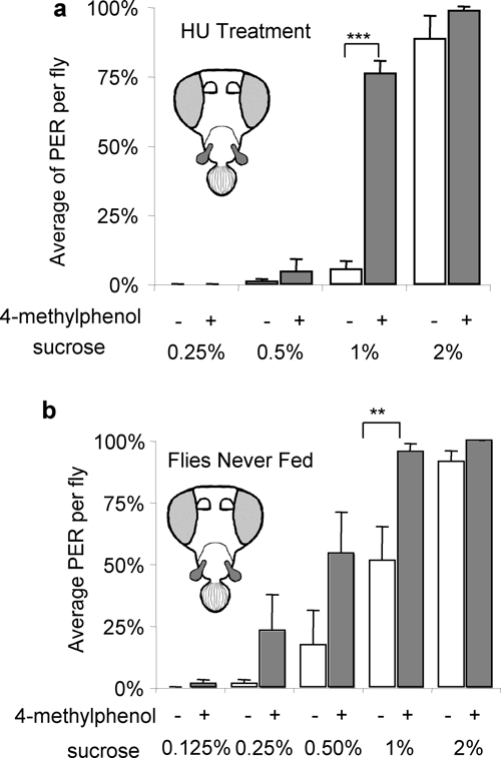
Effect of HU treatment on taste enhancement. Flies were treated with HU at early larval stage. a) Newly eclosed flies were collected before they can feed, and tested. b) Wild type Ant ^−^, MP^+^flies were used. n = 7 to 14

The mushroom body (MB) is involved in odor-induced learning [27]–[29]. It has also been shown that projection neurons connect the MB and the antennal lobe [15]. By treating early larvae with hydroxyurea (HU), the MB can be drastically reduced without harming the fly's ability to smell. These HU-treated flies still exhibited taste enhancement induced by 4-methylphenol (Fig. 4a, Figure S1).

It has been reported that some type of olfactory memory traces can be stored in the projection neurons independent of the MB [42]. Although the tested odors do not exist in the fly food at concentrations used, the flies might have somehow associated odors and taste through normal consumption of the medium. In order to see if this is the case, newly eclosed flies were tested in the same behavior assay. These flies had never encountered food as adults, so there are fewer possibilities to associate smell and taste. The results from these naive flies were similar to normally fed flies (Fig. 4b).

There is still a possibility that flies learned the links between odors with taste during larval stage, and retained that information in a mushroom body-independent manner. There is a study demonstrating that *Drosophila* larvae reared with peppermint scented food would prefer the same odor as adult after eclosion [43]. The only way some of the presented odors could have existed in the fly medium is through fermentation. But this is very unlikely as since the medium contains antifungal reagents, and all flies or larvae did not spend more than 4–5 days in the same vial (see material and methods). The olfactory receptors that detect 4-methylphenol in the maxillary palp are *Or71a* and *Or46a* [36], but neither of these receptors are expressed in larvae [44]. Inactivate yeast is a major component of the medium, but yeast odor did not have any effect on PER to sucrose (data not shown).

**Figure 5:**
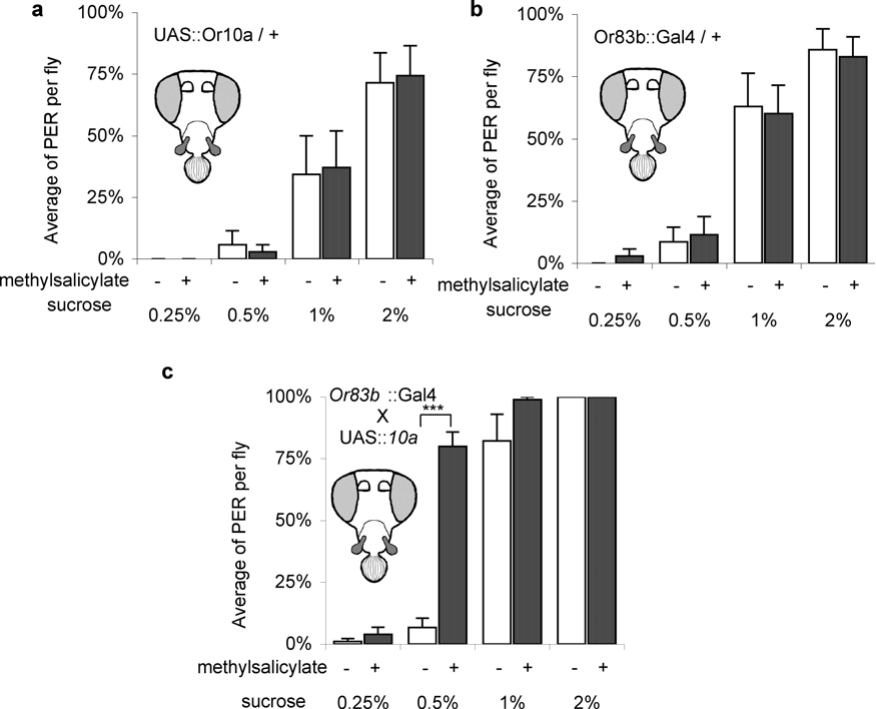
Introducing a foreign receptor in the maxillary palp. Effect of methyl salicylate on taste enhancement in a) *UAS::Or10a*/+, b) *Or83b::Gal*4 /+ line and c) the offspring from the cross *UAS::Or10a*×*Or83b::Gal4*. The threshold for sucrose was different in the three lines. But the significant point was still at the sub-threshold concentration. Ant ^−^, MP^+^flies were used in all experiments. n = 7 to 14

If the OSNs in the maxillary palp are hard-wired to a “taste enhancer circuit”, it should be possible to change the fly's behavior by introducing a foreign olfactory receptor in to the circuit. An antennal olfactory receptor *Or10a* was used for this purpose. This receptor responds to methyl salicylate, an odor that does not elicit responses from any of the 6 OSNs in the maxillary palp [32], [45]. The promoter region of *Or83b* was used to drive expression of *Or10a* in the maxillary palp. Once again, the effect of ectopic expression in the antenna by *Or83b* promoter can be ignored as since the antenna were removed. The flies expressing *Or10a* in the maxillary palp displayed taste enhancement by methyl salicylate at the sub-threshold sucrose concentration, while the parental lines did not (Fig. 5a, b, c).

### The function of maxillary palp is conserved in other fly species

**Figure 6:**
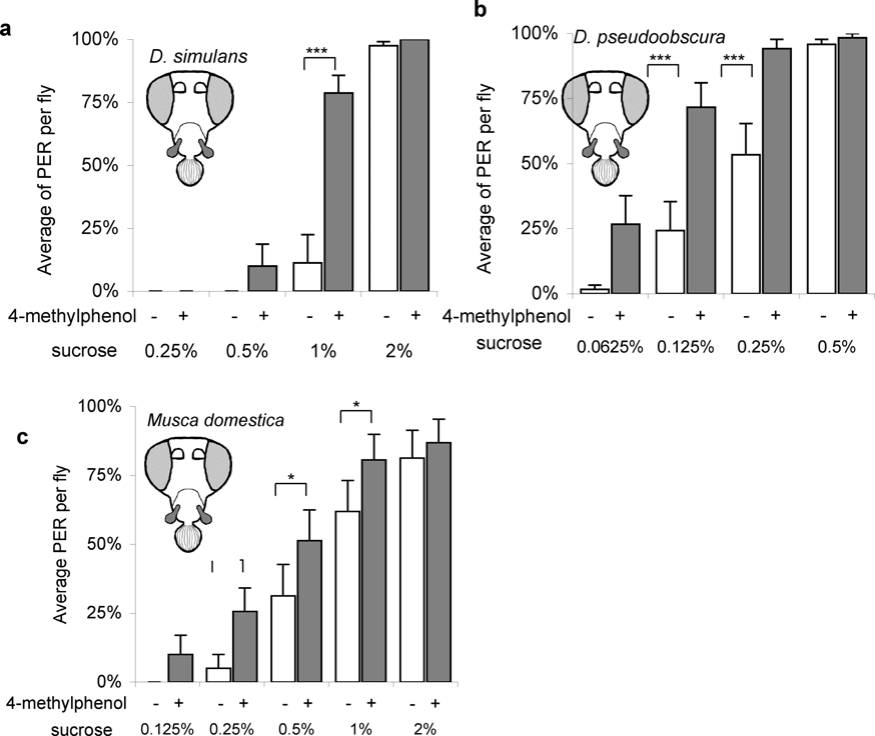
Taste enhancement in a) *D. simulans*, b) *D. pseudoobscura*, c)*Musca domestica*, all without antenna (Ant ^−^, MP^ +^). n = 7 to 14

Many other fly species have morphologically similar maxillary palps. Do they function as taste enhancers in those species as well? First *Drosophila simulans*, a *D. melanogaster* sibling species that diverged approximately 2.5–3.4 million years ago [46], was tested. The effect of 4-methylphenol on taste in *D. simulans* was very similar to that in *D. melanogaster* (Fig. 6a). In order to demonstrate how long this function has been conserved, *D. pseudoobscura*, a more distantly related species, was tested. *D. pseudoobscura* diverged from the *D. melanogaster* lineage 25–55 million years ago and the mean amino acid identity of these two species is 77% for all gene pairs [47]. Even with such a level of divergence, the gene expression pattern of some olfactory receptor genes in the maxillary palp are very similar in these two species, indicating the possible functional conservation of the organ [36]. The sucrose PER threshold in *pseudoobscura* was much lower (0.25–0.5%) than that of *melanogaster* (1–2%). Even with such a large difference in sensitivity to sucrose, taste enhancement by 4-methylphenol was also observed in *pseudoobscura* (Fig. 6b). I tested another species even more distantly related to *D. melanogaster*, *Musca domestica*, the housefly. *Musca domestica* diverged from the *Drosophila* lineage in the middle or early Cretaceous period, 100–140 million years ago [48], [49]. Despite the significant difference in size, morphology and ecological niche between housefly and fruit fly, taste enhancement through the maxillary palp by 4-methylphenol was observed in this species as well (Fig. 6c). Thus all the tested fly species displayed this phenomenon.

## Discussion

### Integration of senses

This study demonstrates that sensory integration can be observed in *Drosophila*. Olfaction modulates the fly's taste behavior through the maxillary palp (Fig. 2). Odor alone does not cause the extension of the proboscis even at high concentration. The sugar neuron in the labellum starts firing at a much lower concentration (<0.3%) than the PER threshold (1–2%) [50]. In between these concentrations is a range that is sweet but not enticing enough to elicit consumption. This decision changes when additional information from the maxillary palp is added. Humans use olfactory information in a very similar fashion. Vanilla is often added to ice cream to enhance the taste, even though vanilla extract itself is not sweet and does not alter the nutritional content. In human it has been shown that schizophrenia patients have an impaired audio-visual integration [51]. In another phenomenon, synesthesia, certain senses are associated together involuntarily [52]. This association does not depend on past experience and many are very difficult to understand for most people, like the taste of a shape or the sound of a color. The significant heritability of schizophrenia and synesthesia suggests that sensory integration may be studied by various genetic tools available *Drosophila*.

### Why are there two olfactory organs?

The olfactory system is highly adaptable to each animal's ecological niche. In insects, size, shape and sometimes even the number of olfactory organs differ between species. In *Drosophila melanogaster* there are two olfactory organs, the third segment of the antenna and the maxillary palp [6]. Homoptera and Hemiptera, the insect groups that contains cicadas and aphids, and some other piercing and sucking insects do not have a maxillary palp at all [53]. In most Lepidoptera, the group that contain moths and butterflies, the maxillary palp is vestigial but the labial palp takes on the role of a secondary olfactory organ [54]. The maxillary palp and the labial palp are both present in the common ancestor of insects [55], so the presence or absence for any of these palps probably reflects the natural selection that occurred during each lineage of the insect's evolution.

Why does *Drosophila* have two separate olfactory organs? Both are covered with hair-like structures called sensilla which harbor olfactory sensory neurons. The maxillary palp is covered with basiconic sensilla, which are also found on the antenna. Olfactory sensory neurons from both olfactory organs send axons into glomeruli in the antennal lobe of the brain. Most of the odors that give responses in the maxillary palp also give responses in the antenna [14], [32]. Thus two olfactory organs share many features in common. There has been a study indicating that maxillary palp is involved in detecting inhibitory compounds from mated females [56]. Detailed electrophysiological analysis of the maxillary palp olfactory neurons did not reveal such function [32], and recent studies have localized olfactory receptor neurons responsible for this type of inhibitory behavior toward mated females to the antennal trichoid sensilla [10], [12]. Another suggested function of the maxillary palp is odor guidance behavior [31]. However, the contribution of the maxillary palp in this behavior is minimal and requires very high odor concentrations for antenna-deprived flies (Ant ^−^, MP ^+^) to display behavioral response level similar to intact flies. Taste enhancement through the maxillary palp described in this study was observed not only by pure chemicals, but also by odor from a natural food source (banana) (Fig. 3d). There are six types of olfactory receptor neurons in the maxillary palp and they are all stimulated by odors found in fruits or fermented materials [32], [57]. Stimulating these neurons by different odors had the same outcome (Fig. 3a).

Specialization to different food sources has been observed in closely related species. *Drosophila melanogaster* and *Drosophila simulans* are cosmopolitan species which share the same habitat, although *D. melanogaster* is more specialized to rotten fruits probably due to higher alcohol tolerance [58], [59]. *D*. *sechellia*, another sibling species, is attracted to and feeds specifically on the fruit *Morinda citrifolia* [60]. The maxillary palp might have played an important role in speciation through food selection since it affects the decision whether to consume it or not. Altering the expression pattern of a single olfactory receptor resulted in a novel behavior (Fig. 5a, b, c). Misexpression of olfactory receptors in the maxillary palp can generate a sub-population that utilizes a new food source, and combined together with a segregation event, this might lead to the emergence of a new species. In fact, a single mutation has been attributed to misexpression of CO_2_ receptors in the maxillary palp; which is a normal characteristic in mosquitoes [61].

It has been shown hat there is a significant genetic variation for olfactory behavior in *Drosophila melanogaster* [62]–[64]. Genes neutral to selection, but carrying the potential of altering animal behavior would be good candidates for initiating speciation.

A well-developed maxillary palp is a common feature of the Diptera group, which includes mosquitoes and houseflies. The positive effect of 4-methylphenol on taste through the maxillary palp occurs in three very different Dipteran species, *D. simulans, D. pseudoobscura* and *M. domestica* (Fig. 6 a, b, c). It has been reported that D-limonene increases PER in the intact Blowfly *Phormia regina* [65]. This indicates that the interaction of taste and smell is a very basic and vital function for the survival of these and probably many other species. If this is conserved within the Diptera group or even beyond, the ability to change feeding behavior by a transgene could be useful for controlling mosquitoes and other pests.

### Maxillary palp as a close range olfactory sensor

The positive effect on taste through the maxillary palp was observed with odors at high concentrations but in a narrow concentration range (Fig. 3 a, b, c). This was surprising because flies would avoid high concentration of most odors, even if it were attractive at low concentration [37]–[39]. Such dual response to a single odor is reasonable as since most chemicals are toxic at high concentrations. Then why is this not the case with the effect of odor on taste through the maxillary palp? The odor intensity depends on not just the concentration, but also the distance from the source. If the purpose of the organ is to evaluate the olfactory information at the very origin, not from a far distance, it should be adapted to the intense degree of odors. The narrow odor concentration range for taste enhancement also supports this theory, since such an olfactory sensor should only be activated at the source, not in the general vicinity. The maxillary palp is located just above the fly's mouthparts, and this is the perfect location for an olfactory organ to assess food that is just about to enter the mouth. Many mammals raise or lower their noses in order to change the focus range for smelling, most notably elephants [66], [67], while *Drosophila* seems to have a separate organ for each purpose.

## Materials and Methods

### Fly stocks

All the flies used in this study were kept and tested at room temperature (21–22°C) unless described otherwise. They were raised on medium containing 10% glucose, 5% inactive yeast, 7% cornmeal, and 0.6% agar. The medium also contains 0.6% propionic acid and 0.1% nipagin as antifungal reagents. The *D. melanogaster* Canton-S stock used in this study was CS-5 described by Monte [39]. Stock *Or83b*::Gal4 and UAS::*Or10a* are described by Hallem [45]. UAS::*shibire^ts1^* stock was a generous gift from Kitamoto [34].

*D. pseudoobscura* and *D. simulans* were obtained from the Tucson *Drosophila* Species Stock Center (Tucson, AZ). *Musca domestica* stock was obtained from Carolina Biological Supply Company (Burlington, NC).

### Effect of odors on taste behavior

The method to investigate the effect of odors on taste is basically a modification of the proboscis extension response assay (PER) [68], [69].

Fly larvae were transferred to new vials to obtain an un-crowded density (100–150 per vial), and eclosed flies were transferred to a fresh medium 0–24 hours after eclosion and were kept for 4–6 days. Prior to the behavioral analysis, flies were anesthetized on a CO_2_ pad in order to remove the antennae and/or the maxillary palps depending on the experimental purpose. *D. melanogaster* were starved for 48±6 hours in an empty vial with a small piece of paper towel, which was kept moist at all times. The starvation time for *D. simulans*, *D. pseudoobscura* and *Musca domestica* was 24–36 hours. I used a starvation condition in which the mortality rate in the vial is around 10–50% for all species tested. Flies were immobilized in a truncated 200 µl pipette tip as described in [70], but with a wider opening which is enough for the fly's head to move and seek for food sources with their proboscis but not with legs (Fig. 1). The pipette tip was placed vertically on a microscope slide and set under a stereomicroscope in a chemical hood with an airflow velocity of 3.2–4.2 cm/s. The ventral side of the fly's head was faced toward the opening of the hood so that air would flow along the ventral to dorsal axis.

A 6 mm wide strip of Kimwipes® (Kimberly-Clark Corp.) paper was twisted into a thread, and pulled apart into small pieces (<1 cm). These small wicks were dipped into sucrose solutions or water and presented to the proboscis by making contact by a thin thread on the tip of the wick. This was repeated ten times with an interval of 3 to 5 seconds apart. For each single fly, the number of responses was divided by the number of contacts made. The PER value was an average of 7 to 14 of these flies in each condition. New wicks were prepared for each condition and for each individual fly. Odors were presented by one of the following methods; a cotton swab held just behind the sucrose solution wick from the fly if the chemical was not soluble in water, or dissolved in water together with sucrose. For the presentation of banana odor, instead of cotton swabs, a small spatula was used to hold paste of overripe banana.

Before applying any samples, the subject flies were tested to see if they were in the proper state for testing. With less starvation, the majority of the flies would not respond even to a high concentration of sugar probably due to the fixation procedure. On the other hand, when starvation is extreme the chance of the fly responding to anything that makes contact or the fly anticipating the approaching object through learning is much higher. First the flies were given a 4% sucrose solution (positive control), which is above the detection threshold. This is to confirm whether i) the starvation time is long enough, ii) the proboscis is not disabled due to any physical damage. Next, a water sample (negative control) was presented to make sure i) the fly did not respond to the positive control because of thirst, ii) the effect of the previous encounter with an “appealing” taste would not have any effect on the following experiment. The negative control was given every time a response to a sucrose solution was observed, and flies were discarded when they responded to it. At a given time, the rate of flies that pass these controls largely varies between vials (0–80%), and the variable that contributes the most is the starvation time relative to the fly's nutritional condition (see Shiraiwa and Carlson [69] for details). The sucrose solutions were given in the following order, 0%, 0.125%, 0.25%, 0.5%, 1%, 2%, 4% (all without odors), 0%, 0.125%, 0.25%, 0.5%, 1%, 2%, 4% (all with odors). A 1/4 dilution of sucrose solution was used for *D. pseudoobscura*.

For statistical analysis, arcsine converted values of the PER were used for paired Student's t-test.

### Temperature control for *shibire^ts1^* expression

A focused light from an additional microscope fiber optic illuminator was used to increase the fly's temperature from room temperature to 30°C. Temperature was monitored by a non-contact infrared thermometer (3M™ Infrared Thermometer IR-500) every minute, and was maintained at 30±1°C by adjusting the output from the illuminator. The shift to 30°C was completed within 2–3 minutes from the start of additional illumination, and the test was conducted after another 2 minutes.

### Ablation of mushroom body by hydroxyurea (HU)

Newly hatched larvae (within 1 hour of hatching) were fed with inactivated yeast paste containing 30–50 mg HU/ml for 3–4 hrs [71]. The larvae were washed briefly and transferred to new medium without HU. The control flies were handled the same way, except for the addition of HU. The effect of HU treatment was confirmed with a mushroom body Gal4 enhancer trap line (Figure S1) [72].

## Supporting Information

Movie S1 The sucrose threshold for this fly was tested prior to filming. The process is described in the subtitle.
(8.19 MB MOV)

Figure S1 HU treatment did not have effect on odor induced taste enhancement. The area surrounded by the white square is where the MB exists. a) A picture of a fly's brain with 203Y×UAS::mcd8GFP. The lobes of the MB are clearly visible. b) A picture of a HU treated fly's brain of 203Y×UAS::mcd8GFP. The size is drastically reduced. 10 samples were observed for each group, and all of them were similar to the ones in the picture. b) HU treated 203Y×UAS::mcd8GFP flies were tested. Odor induced taste enhancement was still present in these flies. Ant -, MP+flies were used in all experiments.
(3.32 MB TIF)
