# Supplementary figures and images for: Multimodal Chemosensory Integration through the Maxillary Palp in Drosophila
Source: PLoS One. 2008 May 14;3(5):e2191. doi: 10.1371/journal.pone.0002191 (PMC2364657; doi:10.1371/journal.pone.0002191)

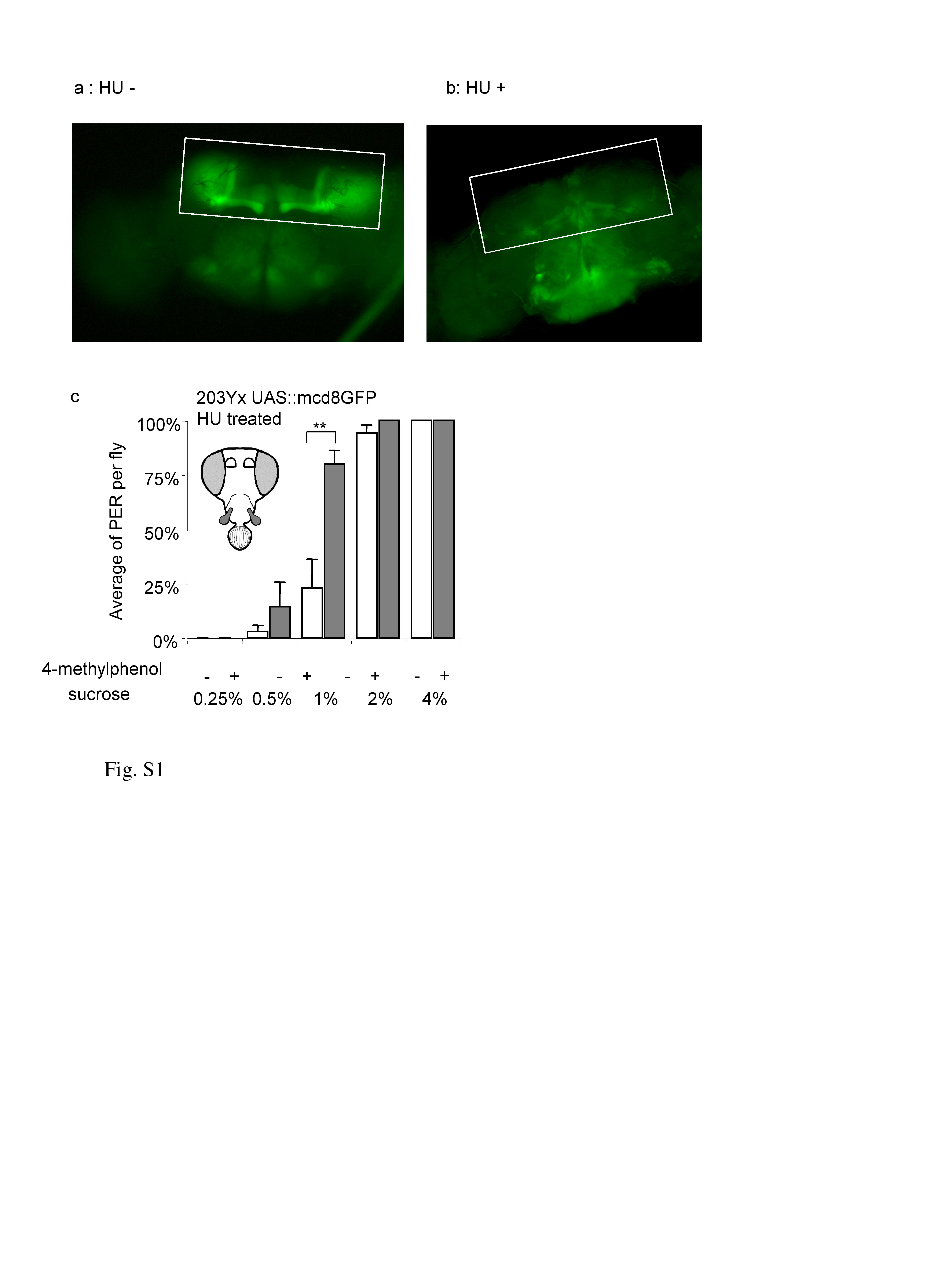

Supplement: Figure S1 — HU treatment did not have effect on odor induced taste enhancement. The area surrounded by the white square is where the MB exists. a) A picture of a fly's brain with 203Y×UAS::mcd8GFP. The lobes of the MB are clearly visible. b) A picture of a HU treated fly's brain of 203Y×UAS::mcd8GFP. The size is drastically reduced. 10 samples were observed for each group, and all of them were similar to the ones in the picture. b) HU treated 203Y×UAS::mcd8GFP flies were tested. Odor induced taste enhancement was still present in these flies. Ant -, MP+flies were used in all experiments. (3.32 MB TIF) [file pone.0002191.s002.tif]
